# Associations of serum uric acid trajectories with hypertension risk: exploring differences between age groups

**DOI:** 10.3389/fcvm.2025.1594142

**Published:** 2025-07-04

**Authors:** Mao Li, Bin Yu, Jia Pan, Chaohui Dong, Honglian Zeng, Shujuan Yang

**Affiliations:** ^1^ Department of Health Management Centre, Clinical Medical College &Affiliated Hospital of Chengdu University, Chengdu University, Chengdu, Sichuan, China; ^2^ West China School of Public Health and West China Fourth Hospital, Sichuan University, Chengdu, Sichuan, China

**Keywords:** hypertension, serum uric acid, changing trajectory, age differences, group-based trajectory modeling

## Abstract

**Background:**

Hyperuricemia is a recognized predictor of hypertension. However, the consistent link between elevated serum uric acid (SUA) levels and hypertension risk across different age groups, especially in males, remains a subject of debate. This study aimed to examine the association between SUA trajectories and the risk of new-onset hypertension, focusing on age-group disparities.

**Methods:**

We conducted a longitudinal study of 4,221 male employees from Southwestern China, initially free of hypertension, over a 4-year period (2018–2021). All employees underwent annual physical examinations each year. Incident hypertension was defined as newly detected blood pressure ≥140/90 mmHg, or the initiation of anti-hypertensive medication. We employed group-based trajectory modeling to identify the trajectory patterns of SUA levels over the study period. Logistic regression was used to assess the association between these SUA trajectories and the risk of developing hypertension. Additionally, subgroup analyses were conducted by stratifying participants into young (<45 years) and middle-aged (≥45 years) groups, with sensitivity analyses conducted using additional cutoffs of 40 and 50 years.

**Results:**

Four distinct SUA trajectories were identified: low (21.18%), moderate-low (45.44%), moderate-high (28.26%), and high (5.12%). The 4-year cumulative incidence of hypertension was 13.2%. Compared to the low SUA trajectory, the high SUA trajectory was independently associated with an increased risk of developing hypertension (OR = 2.22, 95% CI 1.43–3.43). The risk of developing hypertension increased significantly with higher SUA trajectories (*P* for trend = 0.003), and this dose-response relationship was modified by age (*P* for interaction = 0.026). In male employees under 45 years, the high SUA trajectory was associated with a higher risk of hypertension (OR = 1.78, 95% CI 1.04–3.07) compared to the low SUA trajectory, while no significant association was found in male employees over 45 years (OR = 2.22, 95% CI 0.89–5.33). Sensitivity analyses showed a significant association in males <40 years (OR = 2.17), a borderline association in those aged 40–49 (OR = 2.07, *P* = 0.053), but none in those ≥50 (OR = 1.22).

**Conclusion:**

Persistently high SUA levels may elevate hypertension risk in males, particularly younger individuals. Age-specific management of SUA may be necessary for hypertension prevention, with early intervention in young men likely yielding greater benefits than in middle-aged men.

## 1 Introduction

Hyperuricemia has emerged as a prevalent public health concern worldwide, particularly among males (1). According to United States data from 2015 to 2016, there is a significant gender disparity in prevalence, affecting 24.7% of men and 5.2% of women (2). Echoing this trend, in China, the 2018–2019 survey reports reveal a pronounced male predominance, with a prevalence of 24.4% among men and a notably lower rate of 3.6% among women (3). This disparity highlights the urgency of addressing hyperuricemia within the male demographic as a critical public health challenge.

Recent statistics indicate a significant rise in the prevalence of hypertension among Chinese adults, increasing from 25.2% in 2012 to 27.5% in 2019 (4). This trend underscores the growing public health concern posed by hypertension. It is well established that complications associated with hypertension are among the leading causes of disability and all-cause mortality globally. Fortunately, hypertension is preventable and controllable, with effective management reducing risks of cardiovascular diseases, stroke, and related mortality (5).

Hyperuricemia is widely recognized as an independent predictor of prehypertension and hypertension development (6–9), often preceding the onset of hypertension (10, 11). An increasing number of studies suggest that elevated serum uric acid (SUA) levels play a direct role in the pathogenesis and natural history of hypertension (12). Despite this recognition, the consistency of the association between elevated SUA levels and the risk of hypertension across different age groups, particularly in males, remains a subject of ongoing debate (13–16). For instance, a Japanese study has indicated that this association was observed only in men under 45 years, but not in those over 45 years (13). In contrast, another study has shown this relationship to be significant for middle-aged men (aged 45–60), with no observable association in younger or older men (14). What accounts for these inconsistencies in the findings? Notably, SUA levels are influenced by various factors (17) and often exhibit significant fluctuations between baseline and follow-up measurements (18, 19). Baseline SUA values may not represent long-term SUA levels. However, most prior studies have relied on single measurements of SUA levels (6, 8, 13, 14, 20–22), possibly neglecting the dynamic variations of SUA that occur over time, thereby potentially introducing bias into the estimations. Trajectory modeling is a technique that utilizes long-term longitudinal data to analyze trends over time (23), potentially addressing the limitations of single SUA measurements in previous studies. In this study, we conducted a 4-year longitudinal study from 2018 to 2021 to track SUA trajectories and investigate their association with the risk of developing hypertension in male employees, as well as to identify potential age-related heterogeneity in this relationship. The research findings are expected to provide a basis for long-term management strategies of SUA in males of different age groups, aiming to reduce the risk of hypertension.

## 2 Methods

### 2.1 Study design and participants

**Figure 1:**
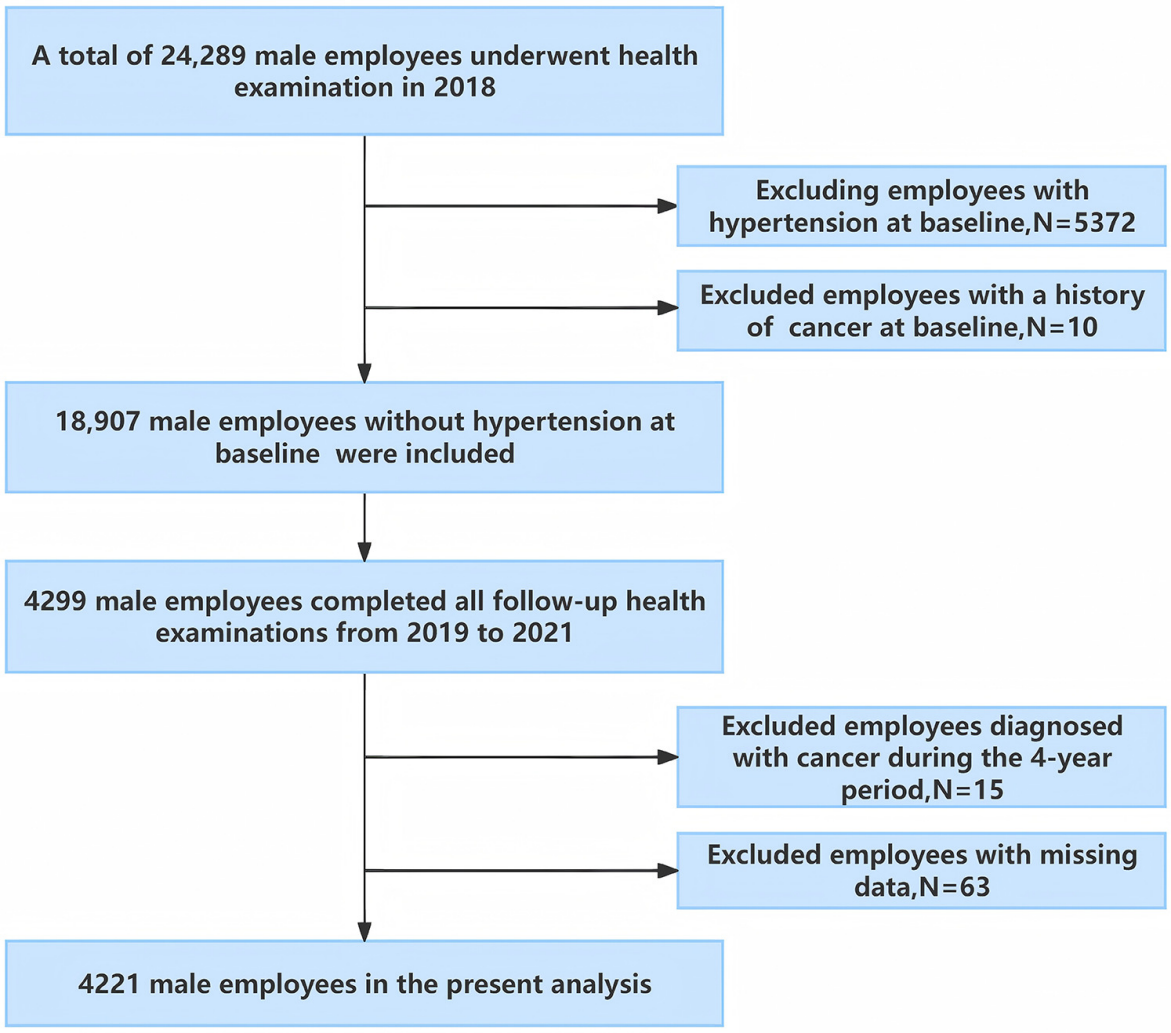
Flow diagram of study enrollment.

This longitudinal study was conducted in southwest China, involving employees from the Chengdu Bureau of the National Railway Administration of China (24). In 2018, 24,289 male employees, aged 20–60 underwent physical examinations at the Affiliated Hospital of Chengdu University and completed a face-to-face survey on demographics and lifestyle behaviors. Individuals with hypertension in 2018 were excluded from the study. Subsequently, annual follow-up examinations were conducted, with 4,299 male employees undergoing annual physical examinations each year from 2019 to 2021. Participants were excluded if they met any of the following criteria: (a) presence of malignant tumors, severe hepatic, or renal insufficiency at baseline or during follow-up exams; (b) incomplete survey or physical examination data (see Figure 1). Ultimately, 4,221 male employees were included in the analysis.

### 2.2 Measurement of hypertension

Annual health check-ups included standardized blood pressure (BP) measurements. A well-trained nurse measured the subject's resting BP in the sitting position after a minimum of 5 min of rest in the morning, utilizing an electronic sphygmomanometer (TM-2655P, A&D Corporation, Japan). Measure three times continuously, with an interval of at least 1–2 min, and take the average as the final systolic blood pressure (SBP) and diastolic blood pressure (DBP). In accordance with the 2024 European Society of Cardiology (ESC) guidelines, incident hypertension was defined as the detection of SBP ≥140 mmHg or DBP ≥90 mmHg, or the initiation of anti-hypertensive medication (25). The occurrence of hypertension during the follow-up was considered as the outcome event.

### 2.3 Measurement of SUA

Annual health check-ups included laboratory tests. Prior to blood collection, all employees fasted for a minimum of 8 h. Subsequently, SUA levels were measured using an automatic biochemical analyzer (AU5800, Beckman Coulter, USA) at the Clinical Laboratory of the Affiliated Hospital of Chengdu University.

### 2.4 Covariates

According to previous studies (8, 26), the factors that affect the association between SUA and hypertension were considered as covariates, including age, marital status, smoking status, drink status, diabetes, family history of hypertension, SBP, DBP, body mass index (BMI), low-density lipoprotein cholesterol (LDL-C), high-density lipoprotein cholesterol (HDL-C), triglycerides (TG), and estimated glomerular filtration rate (eGFR). In this study, face-to-face surveys conducted by uniformly trained physicians collected data from employees on basic demographic information (including age, gender, and marital status), health behaviors (including smoking and alcohol intake), chronic disease history (such as hypertension and diabetes), medication use, and family history of hypertension. Current smokers were defined as those who have smoked at least 100 cigarettes in their lifetime and have been smoke-free for less than a year (27). Current drinkers were defined as subjects who drank at least once a month, and abstinence from alcohol for more than a year was defined as quit drinking. Laboratory assessments, including fasting plasma glucose (FPG), total cholesterol (TC), LDL-C, HDL-C, TG, and serum creatinine (Scr), were conducted. Diabetes was defined by a self-reported history of diabetes, usage of anti-diabetic medicine, or FPG ≥7.0 mmol/L, according to the criteria of the World Health Organization (WHO) (28). The eGFR was calculated using the Cockcroft-Gault equations (29).

### 2.5 Statistical analysis

R (version 4.2.3) was utilized for conducting the statistical analysis. Firstly, group-based trajectory modeling (GBTM) was employed to delineate the trajectory patterns of SUA from 2018 to 2021 among the study cohort. The R package LCTM tools, accessible at [https://github.com/hlennon/LCTMtools], was utilized to establish the GBTM (30). The number of SUA trajectory groups was determined based on the following criteria: (1) the minimization of the Bayesian Information Criterion (BIC); (2) an average posterior probability assignment (APPA) greater than 70%; and (3) a minimum of 5% membership in each trajectory group (23, 31).

Secondly, we presented baseline data based on SUA trajectory groups. Differences in demographic characteristics, lifestyle information, laboratory indicators were analyzed by one-way ANOVA (when the data met normal distribution) and Kruskal–Wallis test (when the data did not meet normal distribution) for continuous variables, and by Chi-square test or Fisher's Exact test, as appropriate, for categorical variables.

Thirdly, we utilized the Cochran-Armitage trend test to analyze the association between SUA trajectory patterns and the cumulative incidence of hypertension. Subsequently, multiple regression models were employed to explore the relationship between these patterns and the risk of developing hypertension, with covariates adjusted step by step. Prior to this, variance inflation factor (VIF) analysis identified multicollinearity. The C statistic was used to assess the predictive accuracy of the models. Additionally, we explored the potential dose-response relationship between SUA trajectory patterns and the risk of developing hypertension by modeling the trajectory groups as a continuous variable in our statistical analyses.

Fourthly, we conducted subgroup and interaction analyses within the adjusted models to identify potential effect modification. Employees were stratified by age (<45 years and ≥45 years) (13, 32), BMI (<24 kg/m^2^ and ≥24 kg/m^2^), current drinking (yes, no), current smoking (yes, no), and family history of hypertension (yes, no). The cross-product term of SUA trajectory patterns and stratified factors was included in the regression model to examine the effect modification.

Finally, we conducted a sensitivity analysis, redefining the age cutoffs at 40 and 50 years old to explore the impact of different age groups on this association (33).

All statistical analyses were conducted in R Studio. Two-sided *P* values below 0.05 were considered statistically significant.

## 3 Results

### 3.1 The trajectory of SUA

**Figure 2:**
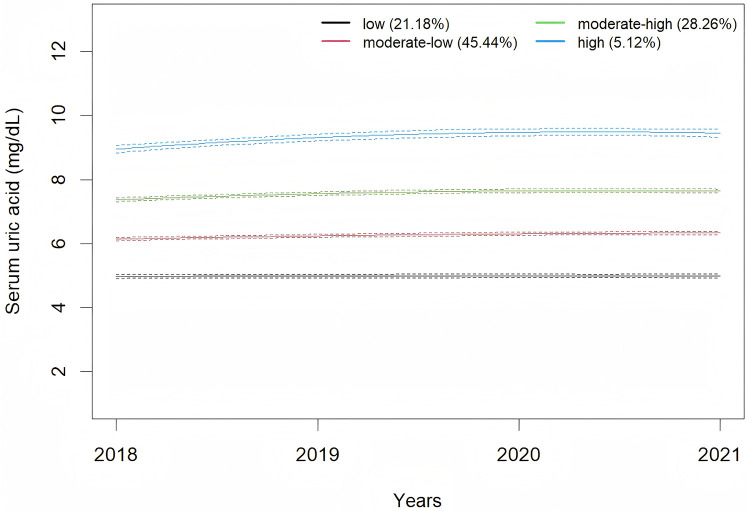
Trajectories of serum uric acid during 2018–2021.

A total of 4,221 male employees were included in present analysis. The average SUA levels from 2018 to 2021 were 6.42 ± 1.30 mg/dl, 6.43 ± 1.37 mg/dl, 6.65 ± 1.38 mg/dl, and 6.55 ± 1.37 mg/dl, respectively. A significant change in SUA levels over time was observed (*F* = 18.92, *P* < 0.001). Employees were classified according to SUA trajectory types, with trajectory models for groups 1–6 compared in sequence. Finally, four SUA distinct trajectories were identified as the best fitted model by GBTM (Table 1). In this model, the minimum proportion of participants in per category was 5.12%, and the minimum APPA for each category was 90.09%. The patterns of the trajectory groups were depicted in Figure 2 and named as follows: low SUA group (*n* = 894, 21.18%), moderate-low SUA group (*n* = 1,918, 45.44%), moderate-high SUA group (*n* = 1,193, 28.26%), and high SUA group (*n* = 216, 5.12%).

**Table 1:** Fitted results of latent class trajectory model for uric acid changes.

Number of trajectories	AIC	BIC	Relative entropy	Category probability (%)	APPA (%)
1	58,246.73	58,272.12	NA	100	NA
2	51,742.17	51,792.95	0.82	62.26/37.74	95.10/94.11
3	48,908.32	48,984.49	0.83	35.70/50.11/14.19	91.99/92.03/92.25
4	47,376.53	47,478.10	0.84	21.18/45.44/28.26/5.12	90.53/90.09/90.68/94.63
5	46,782.12	46,909.08	0.81	13.24/37.19/14.74/32.29/2.53	88.27/86.25/87.88/86.68/94.97
6	46,419.56	46,571.91	0.81	23.08/3.77/37.19/25.44/8.55/1.97	86.55/86.51/84.91/85.75/87.62/92.40

AIC, Akaike information criterion; BIC, Bayesian information criterion; APPA, average posterior probability assignment; NA, when the trajectory quantity is represented by group 1, or when the probability of a particular category is 0 within multiple trajectory groups, there is no corresponding value for the average posterior probability.

### 3.2 Baseline data of different SUA trajectory groups

Table 2 shows the baseline characteristics stratified by SUA trajectory groups. Significant differences were observed across the SUA trajectory groups in age, BMI, SBP, DBP, marital status, prevalence of diabetes, SUA, eGFR, FBG, TC, HDL-C, and TG (*P* < 0.05).

**Table 2:** Baseline demographic clinical characteristic according to serum uric acid trajectories.

Baseline characteristic	Total (*n* = 4,221)	Low (*n* = 894)	Moderate-low (*n* = 1,918)	Moderate-high (*n* = 1,193)	High (*n* = 216)	*P*-value
Demographics						
Age (years), mean ± SD	36.18 ± 10.25	39.38 ± 10.01	36.58 ± 10.24	34.08 ± 9.80	30.99 ± 9.08	<0.001
Married (%)	3,405 (80.7)	775 (86.7)	1,560 (81.3)	924 (77.5)	146 (67.6)	<0.001
Lifestyle behaviors						
Alcohol consumption, *n* (%)						0.058
Never	1,589 (37.6)	370 (41.4)	720 (37.5)	427 (35.8)	72 (33.3)	
Current	2,610 (61.8)	520 (58.2)	1,185 (61.8)	763 (64.0)	142 (65.7)	
Past	22 (0.5)	4 (0.4)	13 (0.7)	3 (0.3)	2 (0.9)	
Smoking status, *n* (%)						0.383
Never	1,688 (40.0)	334 (37.4)	783 (40.8)	485 (40.7)	86 (39.8)	
Current	2,400 (56.9)	538 (60.2)	1,072 (55.9)	666 (55.8)	124 (57.4)	
Past	133 (3.2)	22 (2.5)	63 (3.3)	42 (3.5)	6 (2.8)	
Clinical variables						
Diabetes, *n* (%)	119 (2.8)	58 (6.5)	46 (2.4)	13 (1.1)	2 (0.9)	<0.001
Family history of hypertension, *n* (%)	613 (14.5)	127 (14.2)	267 (13.9)	180 (15.1)	39 (18.1)	0.375
BMI(kg/m^2^), mean ± SD	23.45 ± 3.20	22.24 ± 2.98	23.25 ± 2.98	24.26 ± 3.18	25.88 ± 3.59	<0.001
SBP(mmHg), mean ± SD	119.28 ± 9.60	117.60 ± 9.74	119.27 ± 9.46	119.93 ± 9.60	122.69 ± 9.03	<0.001
DBP(mmHg), mean ± SD	79.13 ± 7.29	78.56 ± 7.43	79.07 ± 7.29	79.50 ± 7.14	79.94 ± 7.35	0.010
SUA(mg/dl), mean ± SD	6.42 ± 1.30	4.98 ± 0.69	6.17 ± 0.68	7.42 ± 0.85	9.04 ± 1.09	<0.001
eGFR(ml/min/1.73 m^2^), mean ± SD	84.90 ± 16.61	81.93 ± 15.35	84.51 ± 16.35	86.69 ± 16.80	90.75 ± 19.98	<0.001
FBG(mmol/L), median(P_25_, P_75_)	4.78 (4.37, 5.18)	4.81 (4.39, 5.25)	4.79 (4.37, 5.19)	4.76 (4.35, 5.12)	4.79 (4.36, 5.16)	0.047
TC(mmol/L), median(P_25_,P_75_)	4.72 (4.15, 5.32)	4.66 (4.10, 5.26)	4.73 (4.15, 5.31)	4.69 (4.15, 5.37)	4.92 (4.34, 5.62)	0.003
LDL-C(mmol/L), median(P_25_, P_75_)	2.47 (2.03, 2.94)	2.42 (1.99, 2.91)	2.48 (2.05, 2.95)	2.46 (2.01, 2.91)	2.60 (2.02, 3.11)	0.054
HDL-C(mmol/L), median(P_25_, P_75_)	1.08 (0.94, 1.25)	1.17 (1.02, 1.34)	1.10 (0.96, 1.27)	1.02 (0.90, 1.18)	0.96 (0.85, 1.11)	<0.001
TG(mmol/L), median(P_25_, P_75_)	1.37 (0.95, 2.08)	1.14 (0.81, 1.61)	1.33(0.92, 1.93)	1.62(1.13, 2.38)	2.11(1.44, 2.99)	<0.001

Continuous variables represented as mean ± SD or median (P_25_, P_75_), categorical variables as *n* (%).

SUA, serum uric acid; BMI, body mass index; SBP, systolic blood pressure; DBP, diastolic blood pressure; eGFR, estimated glomerular filtration rate; FBG, fasting blood glucose; TC, total cholesterol; LDL-C, low-density lipoprotein cholesterol; HDL-C, high-density lipoprotein cholesterol; TG, triglycerides; P_25_, 25th percentile; P_75_, 75th percentile.

### 3.3 Association of SUA change trajectories and hypertension development risk

The cumulative incidence of hypertension among all participants from 2018 to 2021 was 13.2%. Cochran-Armitage trend test showed that the cumulative incidence of hypertension was significantly elevated as SUA changing trajectory increased (Table 3, *P* for trend < 0.001). Prior to conducting multivariable logistic regression models (models 1–3, Table 3), multicollinearity diagnostics identified a collinearity issue between total cholesterol (TC, VIF = 10.52) and low-density lipoprotein cholesterol (LDL-C, VIF = 8.92). Based on subject-matter expertise, TC was excluded from the models to address this issue. Model 3 exhibited a good discriminatory power with a C statistic of 0.77 (95% CI 0.75–0.79). Compared to the low SUA trajectory group, the odds ratios (ORs) with 95% confidence intervals (CIs) for developing hypertension were 0.94 (0.72–1.23) for the moderate-low SUA group, 1.15 (0.85–1.56) for the moderate-high SUA group, and 2.22 (1.43–3.43) for the high SUA group. Furthermore, a significant trend of increasing risk of developing hypertension was observed with escalating SUA trajectories in all three models (*P* for trend < 0.01).

**Table 3:** The association between serum uric acid trajectories and risk of developing hypertension.

Trajectory	Total	Cumulative incidence of hypertension	Model 1		Model 2		Model 3	
			OR(95%CI)	*P*-value	OR(95%CI)	*P*-value	OR(95%CI)	*P*-value
Low	894	11.86%	1 (ref)		1 (ref)		1 (ref)	
Moderate-low	1,918	11.89%	1.00 (0.79, 1.29)	0.981	1.18 (0.92, 1.52)	0.204	0.94 (0.72, 1.23)	0.627
Moderate-high	1,193	14.17%	1.23 (0.95, 1.59)	0.123	1.71 (1.30, 2.24)	<0.001	1.15 (0.85, 1.56)	0.369
High	216	25.00%	2.48 (1.71, 3.57)	<0.001	4.41 (2.97, 6.52)	<0.001	2.22 (1.43, 3.43)	<0.001
*P* for trend		<0.001		<0.001		<0.001		0.003

Model 1: crude model.

Model 2: adjusted for age.

Model 3: adjusted for age, diabetes mellitus, family history of hypertension, marital status, alcohol consumption and smoking status, BMI, SBP, DBP, eGFR, LDL-C, HDL-C, and TG at baseline.

Ref, reference; OR, odds ratios; CI, confidence intervals.

### 3.4 Subgroup and interaction analysis

Table 4 presents the subgroup analysis results. Age-stratified subgroup analyses indicated that the high SUA trajectory was associated with a higher risk of hypertension (OR = 1.78, 95% CI 1.04–3.07) in male employees under 45 years of age, compared to a low trajectory. In contrast, no significant association was observed in male employees aged 45 years and older (OR = 2.22, 95% CI 0.89–5.33). Interaction analyses revealed that the dose-response relationship between SUA trajectories and hypertension development risk was modified by age (*P* for interaction = 0.026).

**Table 4:** Subgroup analyses for the association between serum uric acid trajectories and risk of developing hypertension.

Subgroup	Odds ratio (95% confidence intervals)^a^				*P*_trend_	*P*_interaction_
	Low	Moderate-low	Moderate-high	High		
Age(years)						0.026
<45	1 (ref)	1.01 (0.68, 1.53)	1.19 (0.79, 1.83)	1.78 (1.04, 3.07)*	0.027	
≥45	1 (ref)	0.87 (0.60, 1.26)	0.93 (0.57, 1.49)	2.22 (0.89, 5.33)	0.591	
BMI (kg/m^2^)						0.348
<24	1(ref)	0.87 (0.61, 1.26)	1.35 (0.88, 2.09)	3.10 (1.40, 6.47)**	0.019	
≥24	1(ref)	1.13 (0.76, 1.71)	1.30 (0.85, 2.02)	2.59 (1.49, 4.51)**	0.002	
Current drinking						0.453
No	1(ref)	1.10 (0.68, 1.83)	1.74 (1.00, 3.06)	3.51 (1.53, 7.88)**	0.002	
Yes	1(ref)	0.87 (0.63, 1.20)	0.96 (0.67, 1.38)	1.86 (1.10, 3.13)*	0.119	
Current smoking						0.767
No	1 (ref)	0.97 (0.62, 1.53)	1.44 (0.88, 2.38)	1.92 (0.89, 4.03)	0.026	
Yes	1 (ref)	0.92 (0.66, 1.30)	1.00 (0.68, 1.47)	2.45 (1.41, 4.22)**	0.039	
Family history of hypertension						0.705
No	1 (ref)	0.94 (0.70, 1.27)	1.23 (0.87, 1.72)	1.98 (1.18, 3.27)**	0.011	
Yes	1 (ref)	0.95 (0.52, 1.76)	0.89 (0.45, 1.76)	3.45(1.36, 8.86)**	0.129	

BMI, body mass index.

Test for trend based on the trajectory groups as a continuous variable.

^a^ Adjusted for age, diabetes mellitus, family history of hypertension, marital status, alcohol consumption and smoking status, BMI, SBP, DBP, eGFR, LDL-C, HDL-C, and TG at baseline, except for the one used for stratification.

* *P* < 0.05.

** *P* < 0.001.

### 3.5 Sensitivity analysis

As illustrated in Table 5, upon redefining the age cutoffs, the high SUA trajectory was found to be independently associated with an increased risk of developing hypertension in male employees under 40 years of age (OR = 2.17, 95% CI 1.05–4.69). In male employees aged 40 to under 50 years, there was a borderline significant association (OR = 2.07, 95% CI 0.97–4.30, *P* = 0.053). However, no significant association was observed in male employees aged 50 years and older (OR = 1.22, 95% CI 0.16–6.74).

**Table 5:** Sensitivity analysis for the association between serum uric acid trajectories and hypertension development risk.

Subgroup	Odds ratio (95% confidence intervals)^a^				*P*_trend_
	Low	Moderate-low	Moderate-high	High	
Age(years)					
<40	1 (ref)	1.11 (0.60, 2.19)	1.49 (0.80, 2.95)	2.17 (1.05, 4.69)*	0.009
40–50	1 (ref)	0.96 (0.68, 1.36)	0.84 (0.56, 1.27)	2.07 (0.97, 4.30)^#^	0.797
≥50	1 (ref)	0.67 (0.35, 1.26)	1.49 (0.68, 3.23)	1.22 (0.16, 6.74)	0.496

Test for trend based on the trajectory groups as a continuous variable.

^a^ Adjusted for diabetes mellitus, family history of hypertension, marital status, alcohol consumption and smoking status, BMI, SBP, DBP, eGFR, LDL-C, HDL-C, and TG at baseline, except for the one used for stratification.

* *P* < 0.05.

^#^ *P* = 0.053.

## 4 Discussion

This study utilized GBTM to assess the trajectories of SUA changes among male employees in Southwest China from 2018 to 2021. The SUA change trajectories were classified into four distinct groups. Notably, higher SUA trajectories were found to be associated with an increased risk of developing hypertension, with this association being modified by age. The association between the high SUA trajectory and the risk of developing hypertension was observed in male employees aged 45 years and older, but not in those younger than 45 years.

In this study, as the SUA trajectories increased, the baseline age exhibited a downward trend. This observation aligns with epidemiological surveys in China, which also indicate a trend toward younger onset of hyperuricemia (34). Additionally, in this study, the baseline SUA level was 6.42 ± 1.30 mg/dl, which is notably higher than the levels reported in previous studies (35, 36). This elevation may be associated with factors such as night shift work, irregular diet, insufficient exercise and alcohol consumption among the occupational population (37, 38). The GBTM demonstrated that moderate-high SUA group and high SUA group exhibited SUA levels above the normal range at baseline, and these elevated SUA levels persisted throughout the follow-up period. This pattern implies that individuals within this occupational cohort with hyperuricemia have not adopted measures to lower SUA levels. Previous studies have also found that the majority of individuals with hyperuricemia have not received effective treatment (39, 40). This may be due to the fact that, aside from gout, the harmful effects of hyperuricemia on human health are relatively covert, resulting in inadequate public recognition and insufficient emphasis on the condition.

Our study demonstrated that, compared to the low SUA trajectory, the high SUA trajectory was associated with an increased risk of new-onset hypertension in male employees. This finding is consistent with the work of Ma et al., who also reported that low-increasing and moderate-increasing SUA trajectories was associated with an elevated risk of hypertension (41). These results underscore the importance of monitoring SUA levels over time to better predict and manage the risk of hypertension.

Our study demonstrated that the relationship between SUA trajectories and the risk of new-onset hypertension was modified by age. Specifically, compared to the low SUA trajectory, the high SUA trajectory was significantly associated with an increased risk of developing hypertension in male employees under 45 years of age. However, this association was not statistically significant in male employees aged 45 and older. This finding is similar to a cohort study of Japanese professional men aged 18–64, which reported a significantly higher risk of hypertension in the highest SUA quartile (≥6.7 mg/dl) compared to the lowest (≤5.1 mg/dl). Notably, this significant association was only observed in individuals under 45 years, not in those over 45 (13). However, a prospective cohort study in Japan, which included 26,442 males aged 18–60 years, demonstrated that the association between elevated SUA levels and hypertension was stronger in males aged ≥40 years than in those aged <40 years (15). To further investigate whether different age cutoffs would influence this association, we redefined the age groups in our study. We found that among male employees under 40 years of age, the high SUA trajectory was independently associated with an elevated incidence of hypertension (OR = 2.17, 95% CI 1.05–4.69) compared to the low SUA trajectory. This association tended to be more pronounced in the age group under 40 years compared to the age group under 45 years. Furthermore, a borderline significant association was observed in male employees aged 40–49 years. In the older age subgroup (50 years and above), no significant association was detected. Similarly, a South Korean community-based prospective cohort study of non-hypertensive participants aged 40–79 years, with a mean follow-up of 3.8 years, showed that hyperuricemia significantly increased the risk of incident hypertension in male employees aged 40–49 years. No significant associations were observed in male employees aged 50–59 years or ≥60 years (33). Additionally, two studies focusing on elderly populations have also found no association between SUA levels and the risk of developing hypertension in men (16, 20). Nevertheless, other studies have reported inconsistent findings, suggesting that SUA is associated with the incidence of hypertension in middle-aged and older men as well (14, 42, 43). The potential reasons for these inconsistencies may include the reliance on a single baseline measurement of SUA in some studies, which may fail to account for the dynamic changes in SUA levels over time and thus introduce potential bias. Moreover, the impact of SUA on hypertension appears to vary across different racial groups. For instance, a study comprising 6,399 individuals aged ≥40 years from the U.S. population, utilizing data from the National Health and Nutrition Examination Survey, demonstrated that the association between SUA levels and the incidence of hypertension in men was significant only among Whites, with no significant associations observed among Blacks, Mexican Americans, or other racial groups (43). Future research should encompass large-scale, multinational prospective cohort studies to further substantiate this perspective.

Currently, the potential biological mechanisms underlying why younger men are more susceptible to the hypertensive effects of elevated SUA remain unclear. Several investigations have suggested that the development of hypertension due to elevated SUA occurs through a two-phase process, with the initial phase showing a particularly strong correlation. In this early period, SUA activates the renin-angiotensin system and suppresses neuronal nitric oxide synthase, resulting in heightened renal renin synthesis and reduced systemic nitrate concentrations, which in turn leads to enhanced vasoconstriction (44, 45). A rat model of hyperuricemia, induced by the uric acid oxidase inhibitor oxonic acid, demonstrated a progressive rise in BP over a period of 2–3 weeks. Notably, the early increase in BP observed in this model can be mitigated by discontinuing oxonic acid, administering uric acid-reducing medications, or inhibiting the renin-angiotensin system (46, 47). In the subsequent phase, the persistent increase in SUA levels triggers a cascade of renal pathophysiological alterations. These include the proliferation of vascular smooth muscle cells, reduced compliance of the renal afferent arterioles, and a significant shift in the pressure natriuresis curve. Collectively, these changes result in a form of hypertension that is marked by salt sensitivity and becomes decoupled from uric acid levels. At this stage, hypertension becomes refractory to urate-lowering therapies (48). The pathogenetic mechanisms of hypertension related to hyperuricemia may be more dominant in the earlier stages of hypertension than in the later stages. Additionally, recent studies have shown that with advancing age, senescent vascular endothelial cells (ECs) accumulate progressively. These senescent ECs undergo phenotypic changes that alter the pattern of expressed proteins as well as their morphological and functional characteristics. These alterations in senescent ECs are associated with vascular dysfunction and impairments, such as aortic stiffness, enhanced inflammation, and dysregulated vascular tone, ultimately increasing the risk of cardiovascular diseases, including hypertension (49). Therefore, in middle-aged and older individuals, the impact of age on BP may predominate, while the influence of SUA on BP may diminish. Consequently, it is imperative to address hyperuricemia in younger individuals promptly to mitigate the risk of future hypertension and potentially revert early-stage hypertension.

While this study provides valuable insights, it also has several limitations that warrant consideration. Firstly, the analysis excluded female employees due to their low representation in the railway industry. Secondly, the study population was restricted to adults aged 20–60, as retired railway workers do not participate in annual health examinations. Future research should further investigate the impact of SUA trajectories on BP in men over 60 years of age. Thirdly, although our model accounted for several known confounders associated with hypertension, the influence of unmeasured confounders, such as dietary habits and physical activity, cannot be completely ruled out. Lastly, the relatively short follow-up period is another limitation of this study.

## 5 Conclusion

Our study confirms that persistent high SUA levels are associated with an increased risk of developing hypertension in males, particularly in younger individuals. Therefore, age-specific management of SUA may be necessary for hypertension prevention, with early intervention in young males likely yielding greater benefits than in middle-aged males.

## Data Availability

The raw data supporting the conclusions of this article will be made available by the authors, without undue reservation.
